# MAK-Net: A Multi-Scale Attentive Kolmogorov–Arnold Network with BiGRU for Imbalanced ECG Arrhythmia Classification

**DOI:** 10.3390/s25133928

**Published:** 2025-06-24

**Authors:** Cong Zhao, Bingwei Lai, Yongzheng Xu, Yiping Wang, Haorong Dong

**Affiliations:** 1 Guangdong Laboratory of Artificial Intelligence and Digital Economy (SZ), Shenzhen 518107, China; zhaocong@szu.edu.cn (C.Z.); 2210273120@email.szu.edu.cn (B.L.); 2 Key Laboratory of Optoelectronic Devices and Systems of Ministry of Education and Guangdong Province, College of Physics and Optoelectronic Engineering, Shenzhen University, Shenzhen 518060, China; xuyongzheng@szu.edu.cn (Y.X.); ypwang@szu.edu.cn (Y.W.); 3 Shenzhen Key Laboratory of Photonic Devices and Sensing Systems for Internet of Things, Guangdong and Hong Kong Joint Research Centre for Optical Fiber Sensors, Shenzhen University, Shenzhen 518060, China; 4 Individualized Interdisciplinary Program, The Hong Kong University of Science and Technology, Hong Kong, China; 5 System Hub, The Hong Kong University of Science and Technology (Guangzhou), Guangzhou 511400, China

**Keywords:** ECG classification, multi-scale feature fusion, attention mechanisms, bidirectional GRU (BiGRU), class imbalance

## Abstract

Accurate classification of electrocardiogram (ECG) signals is vital for reliable arrhythmia diagnosis and informed clinical decision-making, yet real-world datasets often suffer severe class imbalance that degrades recall and F1-score. To address these limitations, we introduce MAK-Net, a hybrid deep learning framework that combines: (1) a four-branch multiscale convolutional module for comprehensive feature extraction across diverse waveform morphologies; (2) an efficient channel attention mechanism for adaptive weighting of clinically salient segments; (3) bidirectional gated recurrent units (BiGRU) to capture long-range temporal dependencies; and (4) Kolmogorov–Arnold Network (KAN) layers with learnable spline activations for enhanced nonlinear representation and interpretability. We further mitigate imbalance by synergistically applying focal loss and the Synthetic Minority Oversampling Technique (SMOTE). On the MIT-BIH arrhythmia database, MAK-Net attains state-of-the-art performance—0.9980 accuracy, 0.9888 F1-score, 0.9871 recall, 0.9905 precision, and 0.9991 specificity—demonstrating superior robustness to imbalanced classes compared with existing methods. These findings validate the efficacy of multiscale feature fusion, attention-guided learning, and KAN-based nonlinear mapping for automated, clinically reliable arrhythmia detection.

## 1. Introduction

Cardiovascular diseases (CVDs) represent a major threat to global public health, with rapidly rising incidence rates fueled by modern lifestyles. As the leading cause of mortality worldwide for decades, CVDs claimed 20.5 million lives in 2021—accounting for nearly one-third of all global deaths, according to the World Heart Report [1]. Cardiac arrhythmias—an important subset of cardiovascular diseases characterized by abnormal electrical activity manifesting as tachycardia, bradycardia, or irregular rhythms—pose significant diagnostic challenges due to their transient nature and morphologically diverse electrocardiographic signatures. The advent of electrocardiography (ECG) by Dutch physiologist Willem Einthoven in the early 20th century revolutionized cardiac diagnostics. Today, ECG remains the gold-standard tool for arrhythmia identification, offering temporal resolutions below 1 ms to capture fleeting electrical disturbances [2].

Traditional ECG interpretation relies heavily on expert analysis, making it susceptible to subjectivity and inefficiency, with misdiagnosis rates reaching up to 15.2% in primary care [3]. Recent advances in deep learning have significantly enhanced automated ECG analysis. Convolutional Neural Networks (CNNs) excel at morphological feature extraction [4], while recurrent architectures like LSTMs and GRUs model temporal dynamics [5,6]. Hybrid CNN-RNN models have demonstrated superior classification performance (F1-scores up to 0.923) by capturing both spatial and temporal patterns.

Prior studies have proposed various deep learning solutions: Acharya et al. developed a nine-layer CNN for arrhythmia detection [7]; Yildirim et al. added residual connections [8]; Hannun et al. introduced a deep residual network trained on a large ECG dataset [9]. Hybrid models, such as CNN-LSTM frameworks [10,11], and attention-enhanced networks [12,13], further improved performance. However, two core challenges persist: (1) performance degradation under class imbalance (F1-score drops of 12–18% for minority classes) and (2) sensitivity to noise and non-stationary waveforms, especially in ambulatory recordings [14,15,16].

Building upon the aforementioned studies, we propose a multi-scale attentive Kolmogorov–Arnold network (MAK-Net)—a novel hybrid network model that leverages multi-scale convolution and attention mechanisms to extract rich feature information from ECG signals. The architecture consists of four main modules:

- Multi-scale CNN Module: Utilizes residual convolution with various kernel sizes (e.g., 1 × 3, 1 × 11, 1 × 21, 1 × 31) to capture both local and global morphological features.
- Attention Module: Integrates an enhanced MLPBlock with an Efficient Channel Attention (ECA) layer that uses adaptive 1D convolutions to generate channel-wise importance weights, balancing global context and local detail while adding minimal overhead.
- BiGRU Module: Employs bidirectional Gated Recurrent Unit (GRU) layers to process ECG sequences in both forward and reverse directions, capturing long-range temporal dependencies and contextual features critical for accurate arrhythmia detection
- Kolmogorov–Arnold Network (KAN) Module: Replaces traditional MLPs to enhance nonlinear mapping.

Furthermore, the framework synergizes focal loss optimization with SMOTE-based oversampling, achieving dual mitigation of data imbalance.

The main contributions of this work are as follows:

- Significant gains in minority-class detection: By integrating multiscale residual convolutions with dynamic attention, MAK-Net delivers state-of-the-art recall and F1-scores on imbalanced ECG datasets, overcoming the low sensitivity of existing approaches.
- Parameter-efficient hybrid attention: Our attention module fuses the partial-convolution pruning of the 1D-conv MLPBlock with lightweight ECA channel weighting to capture both local waveform details and global context with minimal overhead, substantially boosting classification accuracy.
- Interpretable nonlinear mapping via KAN: The KAN module replaces traditional MLP weight matrices with edge-based B-spline activation functions, yielding compact symbolic representations that enhance accuracy, interpretability, and cross-domain adaptability [17].
- Robust class-imbalance mitigation: We jointly apply SMOTE oversampling and focal loss (γ = 2.0) to balance training distributions and emphasize hard examples, resulting in markedly improved performance on under-represented arrhythmia classes.

The structure of this paper is organized as follows: Section 2 provides a comprehensive overview of the MAK-Net architecture, details the preprocessing steps applied to the MIT-BIH dataset, and outlines the evaluation protocols employed. Section 3 presents a comparative analysis of our results against state-of-the-art methods. Finally, Section 4 discusses the clinical implications of our findings and offers concluding remarks.

## 2. Proposed Method

**Figure 1:**
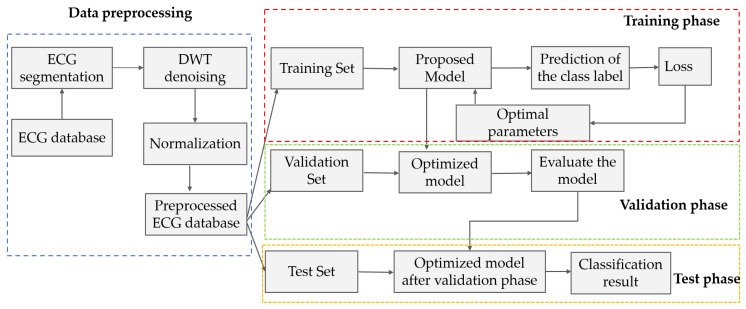
Overview of the MAK-Net ECG classification pipeline.

The comprehensive workflow for ECG signal classification using the MAK-Net model is depicted in Figure 1. The architecture comprises four sequential stages: (1) Data Preprocessing: Raw ECG signals undergo segmentation, denoising through wavelet decomposition and bandpass filtering, and normalization via z-score normalization. Subsequently, the dataset is partitioned into training, validation, and test sets; (2) Training Phase, Model parameters are updated by minimizing the loss function using the training set in a subject-independent (inter-patient) setting; (3) Validation Phase: Hyperparameters (e.g., learning rate, network depth) are optimized to determine the optimal configuration; and (4) Testing Phase: The final performance and generalization capability are evaluated on the test set.

### 2.1. ECG Preprocessing

**Figure 2:**
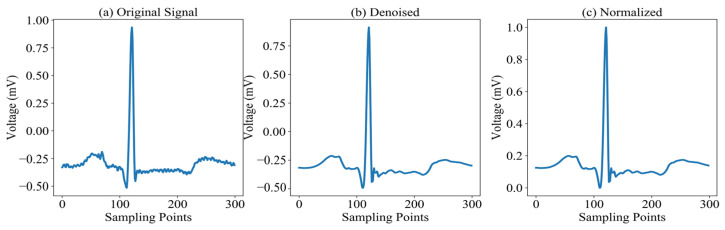
ECG preprocessing pipeline and results: (**a**) Raw ECG segment showing baseline wander and high-frequency noise; (**b**) Denoised signal after discrete wavelet transform; (**c**) Final normalized output using min–max scaling, ready for feature extraction.

In the preprocessing stage, raw ECG signals are first segmented by extracting 150 sampling points on either side of each R-peak to preserve complete waveform morphology. To mitigate noise sources—such as baseline wander, power-line interference, and motion artifacts from muscle activity—the segmented signals undergo denoising before any further processing, thereby improving the accuracy and robustness of downstream feature extraction and classification. In this work, we employ the Discrete Wavelet Transform (DWT) [18], which offers multiscale decomposition, precise time–frequency localization, and energy concentration properties, along with computational efficiency ideally suited for non-stationary biomedical signals [19]. By effectively suppressing noise while preserving diagnostically critical morphological features (as shown in Figure 2b), DWT enhances overall signal quality and boosts classification performance.

The final preprocessing step normalizes the denoised ECG signals to a common amplitude range, thereby accelerating model convergence and improving downstream performance. While various scaling techniques exist—such as min–max normalization and z-score standardization—this study adopts min–max normalization to rescale signal amplitudes to the [0, 1] interval. The normalized signals are shown in Figure 2c, and the transformation is defined in Equation (1):

(1) $$X_{new}=\frac{X-X_{min}}{X_{max}-X_{min}}$$

where *X* denotes the original signal amplitude, *X_min_* and *X_max_* are the minimum and maximum values within the segment, and *X_new_* is the normalized amplitude. Min–max normalization has been shown to eliminate amplitude offsets, standardize feature scales, and significantly speed up gradient-based optimization in ECG classification models [20].

### 2.2. MAK-Net Architecture

**Figure 3:**
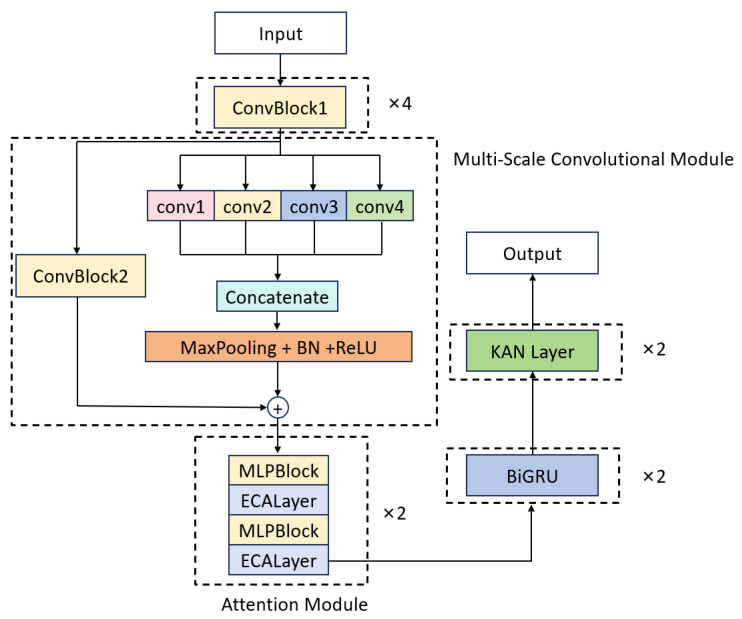
MAK-Net architecture: A ConvBlock for initial feature extraction, followed by multiscale residual convolutions, an attention module, bidirectional GRUs, and a KAN module, leading to the classification output.

Figure 3 illustrates the MAK-Net architecture, which integrates four core modules in sequence: (1) a multi-scale convolutional module with residual connections and parallel kernels (1 × 3, 1 × 11, 1 × 21, 1 × 31) to capture both fine-grained P-wave details and broader RR-interval patterns [21]; (2) an attention module employing channel–spatial reweighting to amplify clinically salient regions such as ST segments and arrhythmic episodes; (3) stacked bidirectional GRU (BiGRU) layers that model long-range temporal dependencies in both forward and backward directions [17]; and (4) a Kolmogorov–Arnold Network (KAN) module that replaces conventional fully connected layers with spline-based activation functions on edges for enhanced nonlinear mapping and interpretability.

Input ECG signals first pass through a four-layer ConvBlock for initial feature extraction, then flow through the multi-scale convolutional module, attention module, BiGRU, and KAN modules in turn, whose complementary spatial, attentional, temporal, and nonlinear representations collectively drive robust arrhythmia classification.

#### 2.2.1. Multi-Scale Convolutional Module

The multi-scale convolutional module draws directly on the InceptionTime paradigm [22], which adapts Inception’s multiplexed convolutions to time-series data by learning multiple filters of varying lengths in parallel. As shown in Figure 3, this module begins with four identical ConvBlock1 units—each comprising a 1D convolution, BatchNorm1d, ReLU activation, and MaxPool1d (kernel = 2, stride = 1, padding = 1)—to perform initial feature extraction. These feature maps then enter a multi-scale fusion block, where four parallel 1D convolutions (kernel sizes 3, 11, 21, 31) capture both fine-grained and broad morphological patterns. The resulting feature maps are combined via weighted averaging, followed by batch normalization, ReLU, dropout (*p* = 0.3), and max pooling to consolidate salient information. Simultaneously, a secondary ConvBlock2 processes the same inputs in parallel, enabling synergistic integration of local and multi-scale representations—an approach shown to improve ECG classification performance in multireceptive-field CNN designs [23].

#### 2.2.2. Attention Module

**Figure 4:**
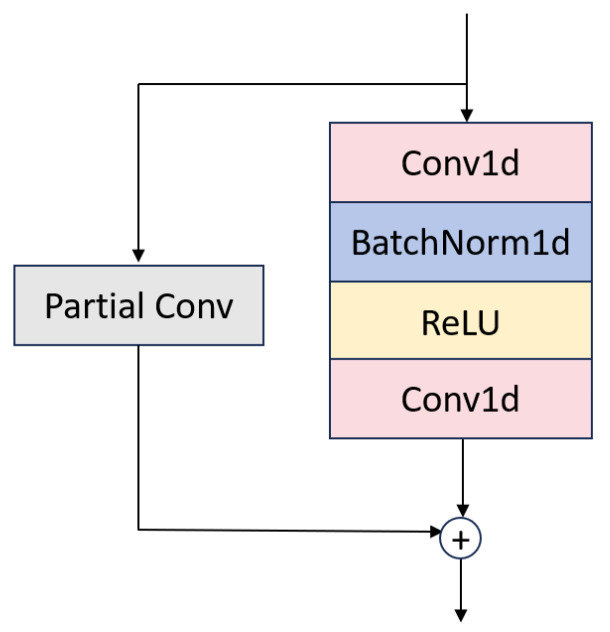
Structure of the MLP Block.

As shown in Figure 3, the attention module alternates two MLPBlock modules with two ECA Layer modules in sequence. Each MLPBlock (Figure 4) employs a dual-branch design: the main branch applies successive Conv1d → BatchNorm1d → ReLU → Conv1d operations to extract local temporal features, while a parallel partial-convolution (Partial Conv) branch performs convolution on only a subset of channels, leaving the remainder unchanged. The two branch outputs are then fused via element-wise addition, leveraging parameter sharing to reduce FLOPs and preserve sequence integrity.

**Figure 5:**
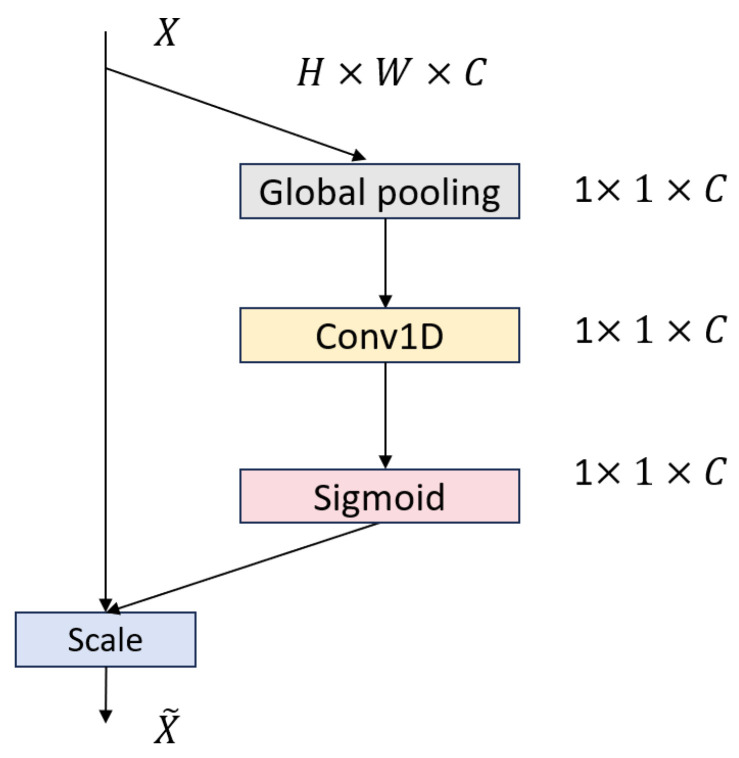
Structure of the ECA layer.

The ECA Layer [24] (Figure 5) implements Efficient Channel Attention by first performing channel-wise global average pooling and then using a single Conv1d (kernel size k determined adaptively) to generate channel weights without any dimensionality reduction. This lightweight recalibration mechanism highlights diagnostically salient channels—such as ST segments and arrhythmic episodes—while suppressing noise, reducing computational complexity by approximately 38% compared to standard self-attention [25].

#### 2.2.3. BiGRU

**Figure 6:**
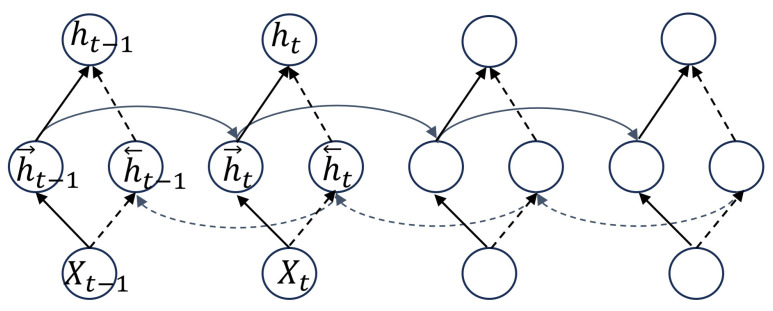
Structure diagram of the bidirectional gated recurrent unit (BiGRU).

As illustrated in Figure 6, the Bidirectional Gated Recurrent Unit (BiGRU) consists of two parallel GRU networks that process the input sequence in forward and backward directions [26]. At each time step *t*, the forward hidden state $\vec{h_{t}}$ and the backward hidden state $\overset{\leftarrow }{h_{t}}$ are computed as:

(2) $$\vec{h_{t}}=GRU(x_{t},\vec{h_{t-1}})$$

(3) $$\overset{\leftarrow }{h_{t}}=GRU(x_{t},\overset{\leftarrow }{h_{t-1}})$$

(4) $$h_{t}=w_{t}\vec{h_{t-1}}+v_{t}\overset{\leftarrow }{h_{t-1}}+b_{t}$$

Here, $x_{t}$ denotes the input vector at time *t*, and $h_{t}$ is obtained by concatenating the forward and backward hidden states. Each *GRU* implements nonlinear gating mechanisms—parameterized by weight matrices $w_{t}$, $v_{t}$ and bias $b_{t}$—to update and reset its hidden state, thereby encoding the ECG signal into a compact representation. By integrating information from both past and future contexts, the BiGRU architecture captures long-range temporal dependencies and richer contextual cues, leading to improved model performance and classification accuracy.

#### 2.2.4. KAN Module

The KAN network, proposed by Liu et al. [27] in 2024, is grounded in the Kolmogorov–Arnold representation theorem [28,29,30], which guarantees that any multivariate continuous function can be decomposed into a finite superposition of univariate continuous functions and addition. Departing from conventional multilayer perceptrons (MLPs) (MLPs) [31,32,33], KAN replaces fixed activation functions at neurons with learnable univariate spline functions on each edge, effectively substituting weight parameters with parametrized activation mappings. A built-in pruning strategy then extracts a compact set of symbolic functions from the trained network, enabling mechanistic interpretability and module-level analysis. Moreover, by aligning its functional basis with the periodic and trend components intrinsic to time-series data, KAN naturally embeds prior domain knowledge into its architecture, boosting performance in scientific and engineering applications.

**Figure 7:**
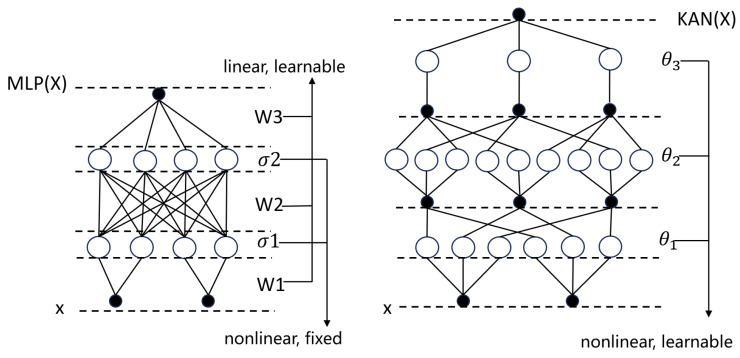
Architecture of the KAN.

As illustrated in Figure 7, whereas an MLP applies fixed nonlinearities after linear summation at each node, KAN nodes perform only linear aggregation, with all nonlinearity arising from the edge-based spline functions—yielding up to 15% gains in accuracy on symbolic regression benchmarks and dramatically enhanced interpretability.

## 3. Experimental Results

### 3.1. ECG Dataset

This study utilizes the MIT-BIH Arrhythmia Database [34], which comprises 48 half-hour excerpts of two-channel ambulatory ECG recordings obtained from 47 subjects studied by the BIH Arrhythmia Laboratory between 1975 and 1979. Subjects include 25 males (32–89 years) and 22 females (23–89 years). Recordings were digitized at 360 Hz with 11-bit resolution over a ±10 mV range; channel 1 is modified lead II (MLII), and channel 2 alternates among V1, V2, V4, and V5. Following Association for the Advancement of Medical Instrumentation (AAMI) recommendations and prior mapping schemes [35], the original 15 beat annotations are consolidated into 5 standard categories—normal (N), supraventricular ectopic (S), ventricular ectopic (V), fusion (F), and unknown (Q)—as detailed in Table 1.

### 3.2. Implementation Detail and Evaluation Metrics

We partitioned the MIT-BIH Arrhythmia Database into training (60%, 65,667 samples), validation (20%, 21,890 samples), and test (20%, 21,890 samples) sets. The training set was used to fit model parameters, the validation set to tune hyperparameters, and the test set to assess final performance. Table 2 summarizes the key hyperparameters used for training MAK-Net. We optimized model parameters using the Adam optimizer with a learning rate of 5 × 10^−4^ over 60 epochs and a batch size of 64. The categorical cross-entropy loss—defined in Equations (5)–(7)—served as the optimization objective.

(5) $$CE(p,y)=\left\{\begin{array}{c} -\log(p),y=1\\ -\log(1-p),otherwise \end{array}\right.$$

Let $p_{t}$ be defined as:

(6) $$p_{t}=\left\{\begin{array}{c} p,y=1\\ 1-p,otherwise \end{array}\right.$$

Therefore, the cross-entropy loss can be formulated as:

(7) $$CE(p,y)=CE(p_{t})=-\log(p_{t})$$

To benchmark performance, we compared MAK-Net against six state-of-the-art methods. Classification efficacy was evaluated using five standard metrics (Equations (8)–(12))—Accuracy, Precision, Recall, F1-Score, and Specificity—to provide a comprehensive assessment of model performance.

(8) $$Accuracy=\frac{TP+TN}{TP+FP+TN+FN}$$

(9) $$Precision=\frac{TP}{TP+FP}$$

(10) $$Recall=\frac{TP}{TP+FN}$$

(11) $$F1-Score=2\times \frac{Precision\times Recall}{Precision+Recall}$$

(12) $$Specificity=\frac{TN}{TN+FP}$$

### 3.3. Classification Performance

**Figure 8:**
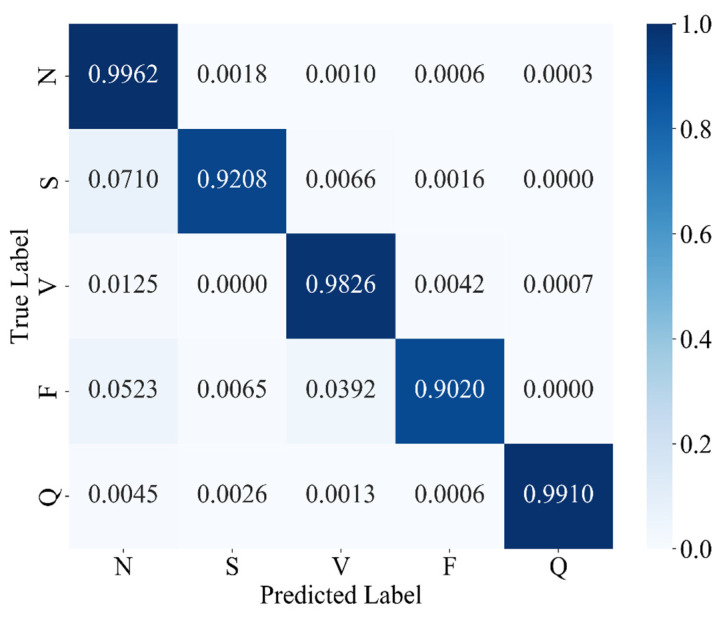
Confusion matrix for MAK-Net with cross-entropy loss.

Under the hyperparameter configuration detailed in Table 2, MAK-Net’s classification results on the test set are summarized in Table 3, with the corresponding confusion matrix depicted in Figure 8. Table 3 reports the standard metrics—accuracy, precision, recall, F1-score, and specificity—for each arrhythmia category, while Figure 8 visualizes true positives, false positives, and false negatives to facilitate a clear understanding of per-class performance and misclassification patterns.

As Figure 8 shows, classes N, V, and Q achieve outstanding accuracies of 99.62%, 98.26%, and 99.10%, respectively, with all associated metrics (precision, recall, F1-score, specificity) exceeding 97.8%. In contrast, classes S and F register lower accuracies—92.08% and 90.20%—and correspondingly reduced F1-scores and recall values, indicating greater misclassification in these minority categories. These results underscore MAK-Net’s robust performance on predominant classes while highlighting areas for further improvement in detecting supraventricular and fusion beats.

### 3.4. Strategies for Mitigating Class Imbalance

The MIT-BIH Arrhythmia Database exhibits a pronounced class imbalance—normal (N), ventricular ectopic (V), and unknown (Q) beats dominate, while supraventricular ectopic (S) and fusion (F) beats are scarce. To counteract this skew, we employ two complementary strategies: (1) Focal Loss [36], which adaptively down-weights well-classified majority samples and emphasizes hard, minority-class examples; and (2) SMOTE [37], which synthetically oversamples underrepresented beats by interpolating between minority instances.

#### 3.4.1. Focal Loss-Based Optimization for Class Imbalance

Focal Loss, first introduced by Lin et al. [36] to address extreme class imbalance in one-stage object detectors, augments the standard cross-entropy loss with a modulating factor and class-balance term to focus learning on hard-to-classify examples. Formally, for a true class probability *p_t_*, it is defined as:

(13) $$FL(p_{t})=-\alpha {\left(1-p_{t}\right)}^{\gamma }\log(p_{t})=\alpha {\left(1-p_{t}\right)}^{\gamma }CE(p_{t})$$

where *p_t_* is the model’s estimated probability for the ground-truth class; *α* ∈ [0, 1] balances the relative importance of positive versus negative samples; *γ* ≥ 0 is the focusing parameter that down-weights well-classified “easy” examples (*p_t_* → 1) and amplifies the loss contribution of misclassified “hard” examples (*p_t_* → 0).

**Figure 9:**
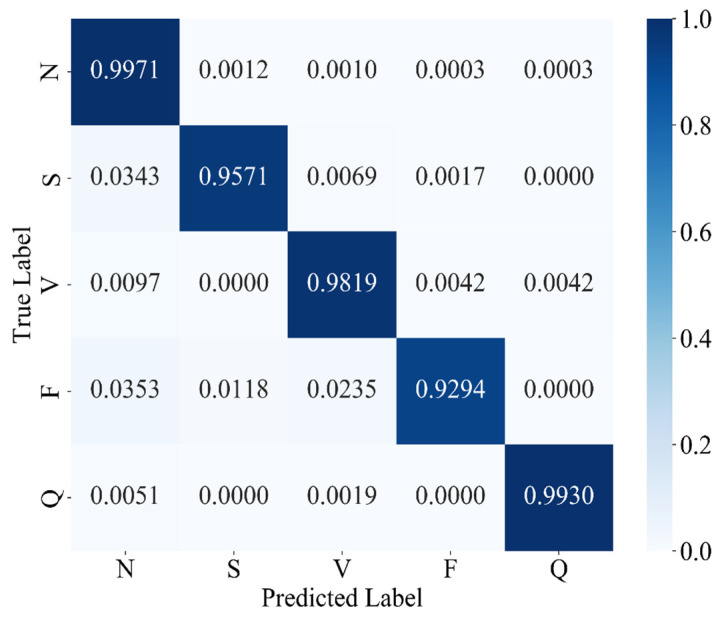
Confusion matrix of MAK-Net optimized with focal loss.

By dynamically modulating the loss contribution of each example, focal loss has been demonstrated to improve model convergence and accuracy in the presence of severe class imbalance [38]. When applied to MAK-Net, it amplifies gradient signals from under-represented arrhythmia classes, thereby increasing sensitivity and reducing bias toward majority classes. Table 4 summarizes the classification performance of MAK-Net optimized with focal loss, and Figure 9 presents the corresponding confusion matrix.

As shown in Table 4, applying focal loss yields marked improvements for the underrepresented S and F classes: MAK-Net achieves accuracies of 95.71% and 92.94% for these classes—representing gains over the previous 92.08% and 90.20%, respectively—while maintaining accuracies above 98% for classes N, V, and Q. These enhancements are further corroborated by the confusion matrix in Figure 9.

To quantify the contribution of each architectural component, we perform a systematic ablation study, in which we iteratively disable or replace key modules and measure the resulting performance drop. Specifically, we (1) substitute the multiscale convolutional block with single-scale variants (kernel sizes 1 × 3, 1 × 11, 1 × 21, 1 × 31) to assess the impact of receptive-field diversity; (2) remove the spatial–channel attention module to evaluate its effect on feature reweighting; (3) omit the BiGRU layers to isolate the importance of bidirectional temporal modeling; and (4) exclude the KAN module to determine its role in nonlinear feature mapping. The observed degradation in accuracy, F1-score, and recall at each step highlights the indispensable roles of multiscale convolution, attention, temporal recurrence, and nonlinear interpretability in achieving state-of-the-art arrhythmia classification.

The ablation results in Table 5 confirm that the multiscale convolutional block consistently outperforms any single-scale variant across all metrics, echoing prior evidence that multiscale filters enhance ECG classification accuracy [39]. Sequentially disabling key modules induces further performance drops: removing the attention block yields a moderate decline, but omitting the BiGRU layers produces the most pronounced decreases in F1-score (−2.53%), precision (−2.22%), and specificity (−2.01%), underscoring the indispensable role of bidirectional temporal modeling [17]. In contrast, exclusion of the KAN module leads to the largest reductions in overall accuracy (−1.21%) and recall (−2.53%), highlighting its power in capturing nonlinear feature interactions crucial for minority-class detection.

#### 3.4.2. SMOTE-Based Synthetic Oversampling for Class Imbalance

SMOTE (Synthetic Minority Over-sampling Technique) is a widely adopted method for addressing class imbalance by generating new, plausible samples for under-represented classes rather than simply duplicating existing ones. Originally introduced by Chawla et al. [37], SMOTE operates in feature space: for each minority-class instance, it identifies its *k* nearest neighbors and then synthesizes new examples by interpolating along the line segments connecting the instance to randomly chosen neighbors. This approach expands the minority-class decision region, mitigates overfitting, and improves classifier sensitivity to rare categories.

In practice, SMOTE proceeds as follows: first, for each minority sample, compute the Euclidean distances to its *k* nearest minority-class neighbors. Next, randomly select one neighbor and generate a synthetic point by taking a convex combination of the two feature vectors using a random weight in [0, 1]. Repeat this process until the minority class reaches the desired sample count. By filling the feature space between existing minority examples, SMOTE balances the training set and enhances the model’s ability to generalize to previously unseen minority-class patterns. To guard against overfitting from synthetic examples, SMOTE was applied only to the training folds during cross-validation, with validation and test sets left entirely unchanged to prevent any data leakage. By isolating synthetic data to the training phase, we ensure that performance metrics reflect the model’s ability to generalize to real ECG segments.

**Figure 10:**
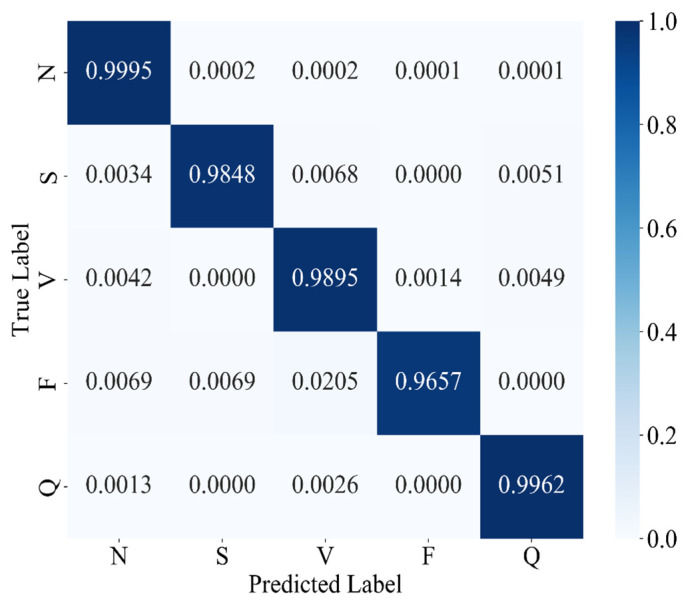
Confusion matrix of MAK-Net trained with SMOTE-oversampled data.

After SMOTE oversampling, each arrhythmia class is represented by approximately 54,170 samples (N: 54,166; S: 54,169; V: 54,168; F: 54,171; Q: 54,171), thereby achieving a fully balanced training set. With class frequencies equalized, we revert to the standard categorical cross-entropy loss to measure discrepancies between predicted probabilities and true labels during training. The classification performance of MAK-Net under this balanced regime is summarized in Table 6, and the corresponding confusion matrix is shown in Figure 10.

As shown in Table 6, MAK-Net trained on SMOTE-oversampled data achieves exceptional performance across all arrhythmia classes: classes N and Q both exceed 99% in accuracy and F1-score, while class V approaches 99% (98.95% accuracy, 98.98% F1-score). Notably, the previously under-represented S and F classes now reach 98.48% and 96.57% accuracy (with F1-scores of 98.81% and 97.24%, respectively), representing substantial gains over their pre-oversampling baselines.

As shown in Table 7, both focal loss and SMOTE substantially boost the accuracies of the minority classes S and F. Focal loss elevates S from 92.08% to 95.71% and F from 90.20% to 92.94%, whereas SMOTE further increases these to 98.48% and 96.57%, respectively. SMOTE also marginally enhances the majority-class accuracies—achieving 99.95% for N, 98.95% for V, and 99.62% for Q—surpassing both the baseline and focal-loss configurations. Therefore, SMOTE-based oversampling proves the most effective strategy for mitigating class imbalance and maximizing overall classification accuracy.

Table 8 compares MAK-Net’s overall metrics—accuracy, F1-score, recall, precision, and specificity—under three optimization strategies: no optimization, focal loss, and SMOTE oversampling. Both focal loss and SMOTE yield marked gains in F1-score, recall, and precision over the unoptimized baseline. SMOTE delivers the largest improvements across all metrics (accuracy: 0.9980, F1-score: 0.9888, recall: 0.9871, precision: 0.9905, specificity: 0.9991). These findings demonstrate that SMOTE-based oversampling more effectively mitigates class imbalance and maximizes overall classification performance compared to focal loss.

### 3.5. Performance Comprasion with Other Reported Methods

As shown in Table 9, MAK-Net outperforms all benchmark methods in accuracy (0.9980), precision (0.9905), F1-score (0.9888), and specificity (0.9991), while maintaining a competitive recall (0.9871) compared to the highest recall achieved by Ahmed et al. [40]. This balanced, state-of-the-art performance reflects several broader insights in ECG arrhythmia classification. First, hybrid architectures that combine convolutional feature extractors with recurrent or attention modules consistently surpass single-model approaches by capturing both spatial and temporal dependencies in ECG signals [41]. Second, multiscale convolutional streams integrated with lightweight attention mechanisms—also as in CNN-BiLSTM-BiGRU hybrids [42]—enhance discriminative power by jointly modeling fine-grained waveform details and global rhythm patterns. Third, embedding interpretable components, such as spline-based activation functions in KAN or hierarchical attention blocks, not only boosts accuracy but also provides mechanistic transparency, a crucial factor for clinical adoption. Together, these trends underscore the effectiveness of MAK-Net’s design in extracting rich, clinically relevant features and delivering robust arrhythmia classification.

Table 10 benchmarks MAK-Net against four canonical deep learning baselines—ResNet, RNN, LSTM, and GRU—each retrained on the MIT-BIH dataset under identical conditions. While these architectures achieve accuracies in the 0.9786–0.9824 range and F1-scores between 0.9003 and 0.9119, MAK-Net attains 0.9980 accuracy and an F1-score of 0.9888, representing gains of 1.56–1.94% in accuracy and over 7.0% in F1-score. The pronounced improvements in both precision and recall underscore MAK-Net’s balanced performance across arrhythmia types. These results indicate that, although standard CNN and RNN variants can capture general morphological or temporal patterns, they struggle with the complex, multiscale dynamics of ECG signals. In contrast, MAK-Net’s tailored integration of multiscale convolutional streams, attention reweighting, bidirectional recurrence, and nonlinear KAN modules enables richer feature extraction and more robust classification across all arrhythmia classes.

## 4. Discussion

The experimental results confirm that MAK-Net delivers state-of-the-art ECG classification performance (accuracy 0.9980; F1-score 0.9888; specificity 0.9991), validating our design choices. The multiscale convolutional streams capture both fine-grained morphological features (e.g., QRS complexes) and broader rhythm patterns, while the ECA attention mechanism dynamically reweights diagnostically salient segments—analogous to a clinician’s focus on ST deviations and P-wave abnormalities [39]. The BiGRU layers encode bidirectional temporal dependencies, essential for detecting arrhythmias that evolve over extended intervals [50]. Furthermore, the KAN module’s learnable spline activations replace fixed nonlinearities, enabling post-hoc symbolic analysis of feature interactions and greatly enhancing interpretability—a critical requirement for clinical adoption [51].

Our ablation and comparative studies also highlight the pivotal role of data-level balancing. SMOTE oversampling outperforms focal-loss reweighting alone, boosting minority-class recall and F1-scores by up to 3.8% over baseline models—consistent with prior findings on class-imbalance remedies in ECG tasks [52]. This underscores that, even with advanced loss functions, equitable class representation in the training set remains indispensable for robust arrhythmia detection.

Clinically, MAK-Net’s near-perfect specificity (≥99.7%) minimizes false alarms for life-threatening conditions such as ventricular fibrillation, while its high recall (≥98.4% for rare beats) ensures reliable detection of critical but infrequent events like supraventricular ectopics. Moreover, the model’s interpretable attention maps and spline-based activations can help clinicians verify that the network’s focus aligns with established diagnostic markers, thereby fostering trust.

Nonetheless, there are still several challenges in further applying the MAK-Net in real clinical use. Generalizability to real-world ECG recordings—often corrupted by motion artifacts, electrode misplacement, and variable sampling rates—must be assessed on larger, more heterogeneous datasets [52]. The hybrid architecture’s computational complexity may also hinder deployment on wearable or edge-computing platforms, motivating future work on model compression, pruning, and quantization to retain performance while reducing inference time [53]. More specifically, we have measured MAK-Net’s computational footprint at 6.11 million parameters and an end-to-end inference time of 1.52 s for a batch of sixty-four ECG segments on a cloud server equipped with an NVIDIA RTX 3090 GPU and 14 vCPU Intel^®^ Xeon^®^ Platinum 8362 CPUs, corresponding to approximately 24 ms per segment. Although this complexity and latency preclude ultra-low-latency continuous monitoring, at this stage, MAK-Net is well suited for offline or semi-real-time applications requiring high diagnostic precision—such as detailed analysis of rare channelopathies (e.g., Brugada syndrome) or refined myocardial ischemia assessments. Finally, demographic biases in training data (e.g., underrepresentation of certain age or ethnic groups) necessitate rigorous fairness evaluations and bias-mitigation strategies before clinical rollout.

Looking forward, the modular nature of MAK-Net invites adaptation to other biosignal domains—such as EEG seizure detection or respiratory monitoring—where multiscale spatiotemporal modeling is equally vital. Incorporating causal inference frameworks could further refine arrhythmia precursor detection, transitioning from correlation-based classification to actionable diagnostic insights. To ensure comparability with the majority of existing studies using the MIT-BIH dataset, we adopted an intra-patient evaluation protocol, which is widely regarded as the standard approach given the dataset’s limited cohort size [54,55]. We acknowledge that inter-patient validation offers a more stringent test of generalizability, and we consider it a valuable direction for future work. In terms of the interpretability of MAK-Net, we have not included explicit visualizations or symbolic analyses of the learned spline activations; our interpretability claims are grounded in the architectural design of KANs, which employ learnable spline-based activation functions on edges, facilitating transparent and adaptable functional mappings [28,29,30]. Thus, we intend to analyze the learned spline functions to extract symbolic representations, which could offer deeper insights into the model’s decision-making process and enhance its clinical utility.

We plan to extend MAK-Net’s validation to larger, publicly available ECG repositories—such as the PTB-XL 12-lead dataset and the CPSC 2018 challenge dataset—to robustly assess cross-center generalizability. In addition, we also plan to evaluate MAK-Net’s robustness using more diverse and noise-prone datasets, such as the MIT-BIH Noise Stress Test Database, which includes recordings with baseline wander, muscle artifacts, and electrode motion artifacts, and the Brno University of Technology ECG Quality Database (BUTQDB), which comprises long-term ECG recordings collected under free-living conditions with varying signal qualities. These evaluations will help assess and enhance the model’s performance in more realistic scenarios. Additionally, in collaboration with cardiologists at the First Affiliated Hospital of Guangzhou Medical University, Guangzhou, China, we are curating a Brugada syndrome cohort, annotated for Type-1 and Type-2 ECG patterns, to evaluate MAK-Net’s sensitivity and specificity on this rare but clinically critical channelopathy. In sum, MAK-Net exemplifies how architectural innovation coupled with data-centric optimization can yield interpretable, reliable AI tools that augment, rather than replace, physician expertise in cardiovascular diagnostics.

## 5. Conclusions

This study introduces MAK-Net, a hybrid deep learning framework that combines multiscale convolutional streams, efficient channel attention, bidirectional GRUs, and a Kolmogorov–Arnold Network layer to deliver state-of-the-art ECG arrhythmia classification—achieving 0.9980 accuracy, a 0.9888 F1-score, 0.9871 recall, 0.9905 precision, and 0.9991 specificity on the MIT-BIH database. By integrating SMOTE-based oversampling with focal-loss optimization, MAK-Net effectively mitigates class imbalance, with SMOTE proving most impactful for minority-class detection. The model’s attention maps and spline-based activations enhance interpretability, aligning network focus with clinical markers, while its lightweight hybrid design suggests feasibility for real-time deployment. Nevertheless, further validation on heterogeneous, noisy ECG recordings and efforts in model compression and fairness assessment are essential before clinical translation. Future work will explore adapting MAK-Net’s modular architecture to other biosignals (e.g., EEG) and incorporating causal inference to advance from correlation to actionable diagnostic insights.

## Figures and Tables

**Table 1:** ECG class mapping using the AAMI standard.

Class	Symbol	Heart Beat	Training Samples	Validation Samples	Test Samples
Normal beat	N	Normal beat	54,171	18,058	18,116
		Left bundle branch block beat			
		Right bundle branch block beat			
Supraventricular premature beat	S	Atrial premature beat	1807	603	616
		Nodal (junctional) premature beat			
		Supraventricular premature beat			
		Aberrated atrial premature beat			
		Atrial escape beat			
		Nodal (junctional) escape beat			
Premature ventricular contraction	V	Premature ventricular contraction	4348	1449	1438
		Ventricular escape beat			
Fusion of ventricular and normal beat	F	Fusion of ventricular and normal beat	483	161	158
Unknown Beat	Q	Pace beat	4858	1619	1562
		Unclassifiable beat			
		Fusion of paced and normal beat			

**Table 2:** Hyper-parameter of the model training.

Hyper-Parameter	Values
Batch size	64
Learning rate	0.0005
Epoch	60
Optimizer	Adam
Loss function	Cross-Entropy Loss

**Table 3:** MAK-Net classification performance (cross-entropy loss).

Class	Accuracy	F1-Score	Recall	Precision	Specificity
N	0.9962	0.9960	0.9962	0.9958	0.9798
S	0.9208	0.9292	0.9208	0.9378	0.9982
V	0.9826	0.9806	0.9826	0.9785	0.9985
F	0.9020	0.8903	0.9020	0.8790	0.9991
Q	0.9910	0.9933	0.9910	0.9955	0.9997
Sum	0.9922	0.9579	0.9585	0.9573	0.9951

**Table 4:** Classification performance of MAK-Net optimized with focal loss.

Class	Accuracy	F1-Score	Recall	Precision	Specificity
N	0.9971	0.9972	0.9971	0.9973	0.9872
S	0.9571	0.9579	0.9571	0.9588	0.9989
V	0.9819	0.9809	0.9819	0.9799	0.9986
F	0.9294	0.9267	0.9294	0.9240	0.9994
Q	0.9930	0.9926	0.9930	0.9923	0.9994
Sum	0.9942	0.9711	0.9717	0.9705	0.9967

**Table 5:** Ablation study results of MAK-Net optimized with focal loss.

**Configuration**	**Accuracy**	**F1-Score**	**Recall**	**Precision**	**Specificity**
Single-scale (1 × 3)	0.9878	0.9662	0.9667	0.9658	0.9826
Single-scale (1 × 11)	0.9867	0.9646	0.9657	0.9636	0.9836
Single-scale (1 × 21)	0.9842	0.9583	0.9637	0.9531	0.9825
Single-scale (1 × 31)	0.9853	0.9545	0.9645	0.9447	0.9835
Without Attention module	0.9871	0.9566	0.9590	0.9543	0.9731
Without BiGRU	0.9821	0.9529	0.9464	0.9596	0.9722
Without KAN	0.9838	0.9485	0.9487	0.9483	0.9627
Full MAK-Net	**0.9942**	**0.9711**	**0.9717**	**0.9705**	**0.9967**

**Table 6:** Classification performance of MAK-Net trained with SMOTE-oversampled data.

Class	Accuracy	F1-Score	Recall	Precision	Specificity
N	0.9995	0.9994	0.9995	0.9994	0.9970
S	0.9848	0.9881	0.9847	0.9915	0.9997
V	0.9895	0.9898	0.9895	0.9902	0.9993
F	0.9657	0.9724	0.9658	0.9792	0.9998
Q	0.9962	0.9942	0.9961	0.9923	0.9994
Sum	0.9980	0.9888	0.9871	0.9905	0.9991

**Table 7:** Per-class accuracy of MAK-NET under different imbalance mitigation strategies.

Method	N	S	V	F	Q
No Optimization	0.9962	0.9208	0.9826	0.9020	0.9910
Focal Loss	0.9971	0.9571	0.9819	0.9294	0.9930
SMOTE	0.9995	0.9848	0.9895	0.9657	0.9962

**Table 8:** Overall performance metrics of MAK-Net under different optimization strategies.

Method	Accuracy	F1-Score	Recall	Precision	Specificity
No Optimization	0.9922	0.9579	0.9585	0.9573	0.9951
Focal Loss	0.9942	0.9711	0.9717	0.9705	0.9967
SMOTE	0.9980	0.9888	0.9871	0.9905	0.9991

**Table 9:** Comparative classification performance of MAK-Net and recent state-of-the-art methods.

Authors	Method	Accuracy	F1-Score	Recall	Precision	Specificity
Yamac et al. [43]	1D-CNN	0.9820	0.9190	0.9370	0.9280	0.9880
Pu et al. [44]	1D-BNN	0.9750	0.9640	0.9790	0.9650	0.9980
Eleyan et al. [35]	CNN-LSTM	0.9740	0.9730	0.9740	0.9730	NA
Li et al. [45]	CNN	0.9810	NA	NA	NA	0.9870
Hammad et al. [46]	Fusion CNN	0.9880	0.9880	**0.9880**	0.9880	0.9880
Ahmed et al. [40]	AAC-FFAHD	0.9880	NA	0.9850	NA	0.9790
This work	MAK-Net	**0.9980**	**0.9888**	0.9871	**0.9905**	**0.9991**

**Table 10:** Performance comparison with baseline models.

Author	Method	Accuracy	F1-Score	Recall	Precision	Specificity
He et al. [47]	ResNet	0.9786	0.9003	0.9039	0.8971	0.9870
Zaremba et al. [48]	RNN	0.9798	0.9097	0.9400	0.8842	0.9911
Hochreiter et al. [49]	LSTM	0.9824	0.9119	0.9325	0.9068	0.9911
Chung et al. [6]	GRU	0.9806	0.9089	0.9385	0.8842	0.9916
This work	MAK-Net	**0.9980**	**0.9888**	**0.9871**	**0.9905**	**0.9991**

## Data Availability

The data may be requested by reaching out to authors through email.

## Abbreviations

The following abbreviations are used in this manuscript:

AAMI	Association for the Advancement of Medical Instrumentation
ACGANs	Auxiliary Classifier Generative Adversarial Networks
BiGRU	Bidirectional Gated Recurrent Unit
CNNs	Convolutional Neural Networks
Conv1d	1-Dimensional Convolution
CVDs	Cardiovascular Diseases
DWT	Discrete Wavelet Transform
ECA	Efficient Channel Attention
ECG	Electrocardiogram
EEG	Electroencephalogram
FLOPs	Floating Point Operations
GRUs	Gated Recurrent Units
KAN	Kolmogorov–Arnold Network
LSTM	Long Short-Term Memory
MIT-BIH	Massachusetts Institute of Technology-Beth Israel Hospital (Arrhythmia Database)
MLII	Modified Lead II
MLP	Multilayer Perceptron
ReLU	Rectified Linear Unit
RNNs	Recurrent Neural Networks
SMOTE	Synthetic Minority Oversampling Technique
